# Most of patients with localized prostate cancer will be treated in the future? | *Opinion: No*

**DOI:** 10.1590/S1677-5538.IBJU.2017.04.03

**Published:** 2017

**Authors:** Ariel A. Schulman, Thomas J. Polascik

**Affiliations:** 1 Division of Urology, Duke University Medical Center, Durham, NC, USA

**Keywords:** Prostatic Neoplasms, Patients, Epidemiology

We are in the midst of a major shift in the diagnosis and management of localized prostate cancer. The prevailing approach of the 1980’s and 1990’s focused on widespread population-based prostate specific antigen (PSA) testing and curative-intent treatment for any detected cancer. The philosophical approach in the most recent decade is now defined by risk-adapted PSA screening, and integration of novel imaging techniques and biomarkers to increase the detection of clinically significant cancer. Concomitantly, we are witnessing the expanding utilization of active surveillance and partial ablation strategies to avoid overtreatment. We believe that continued development in each of these areas will continue to decrease the number of patients with localized disease treated with traditional whole gland surgery or radiation in the future.

## Decreased screening practices

The incidence of prostate cancer rose steadily in the 1980’s and exhibited a sharp increase in the early 1990’s following the clinical integration of PSA as a screening test for prostate cancer (1). But subsequent concerns about the overdiagnosis of indolent disease and side effects associated with treatment raised questions about the benefit of widespread population screening. The United States Preventive Services Task Force (USPSTF) initially raised concerns about PSA screening in 2008 and in 2012 issued a recommendation against any PSA screening for prostate cancer (Grade D) (2). This recommendation was based in great part on results from the Prostate, Lung, Colorectal and Ovarian (PLCO) Cancer Screening Trial that did not show a cancer-specific mortality benefit in PSA screened men. It should be noted that subsequent detailed analysis of the trial has demonstrated that over 80% of patients in the ‘control’ group underwent ≥2 PSA tests within 3 years before entry into the trial contaminating the study findings (3). Nevertheless, the USPSTF recommendation has had a significant impact on screening practices in the United States.

Jemal et al. found meaningful decreases in both prostate cancer screening practices and incidence rates in 18 Surveillance, Epidemiology, and End Results (SEER) registries from 2005 through 2012 (4). The percentage of men 50 years old and over who reported PSA screening decreased from 40.6% in 2008 to 30.8% in 2013 and the incidence of prostate cancer in men 50 and over declined from 540.8 per 100.000 men in 2008 to 416.2 per 100.000 men in 2012. At the same time, there is some evidence suggesting that rates of metastatic disease at diagnosis are increasing following decreased screening. Hu et al. reviewed SEER data from 2004 to 2013 and noted an increase in both the proportion of men presenting with intermediate and high risk disease (46.3% to 56.4%, p <0.1) as well as an increase in men presenting with distant metastases from 2.7% to 4.0% across this time period (5). It appears that changing practices are also impacting curative treatments with a 16.2% decrease in urologist radical prostatectomy volume between 2009 and 2016 (6).

Thus, recent controversies surrounding screening practices, at least in the United States, have had a negative impact on the proportion of men being screened, the proportion of men diagnosed with localized disease and the number of men undergoing radical prostatectomy. One corollary to this phenomenon is that men with low risk disease identified through screening will likely not be subjected to radical treatment, as has been the standard practice in the past.

## Improved risk stratification

The last decade has also been notable for the proliferation of biomarkers and the integration of multiparametric magnetic resonance imaging (mpMRI) into the initial evaluation and management of men with localized prostate cancer.

Ahmed et al. have recently reported results from the Prostate MR Imaging Study (PROMIS) that compared the performance of mpMRI and transrectal ultrasound (TRUS) guided 12-core biopsy against a reference transperineal template mapping (TPM) biopsy in biopsy-naïve men with clinical suspicion of prostate cancer (7). In the analysis of 567 men, mpMRI was significantly more sensitive than TRUS biopsy (93% versus 48%, p <0.0001) for detection of clinically significant cancer. The negative predictive value of mpMRI was 89.2% compared to 73.7% for TRUS biopsy. Based on these findings the authors suggested that pre-biopsy mpMRI significantly decreases the diagnosis of clinically insignificant prostate cancer and may be considered as a triage test in selecting men for biopsy. The integration of image-guided fusion biopsy also improves the histologic detection of clinically significant prostate cancer. Valerio et al. performed a systematic review of the efficacy of mpMRI targeted biopsies and found that targeted biopsies detected significantly more clinically significant cancers compared to standard TRUS biopsy (33.3% versus 23.6%). Thus, recent evidence suggests that mpMRI is improving the visual and histologic detection of clinically significant cancer, while likely decreasing the diagnosis of clinically insignificant cancer. The current obstacle to widespread adoption of mpMRI and fusion biopsy is cost, but with time, expenses tend to decline enabling these technologies to become more readily available. Further, we expect that with progress, additional imaging technologies will be introduced that provide both anatomical and functional characterization of cancers, further distinguishing tumors that do not require treatment.

There have also been meaningful advances in the role of biomarkers to optimize screening and prostate cancer detection. Multiple blood, urine and tissue assays are now available to guide both screening and treatment recommendations. The serum based Prostate Health Index® (PHI) and 4Kscore® have both demonstrated pre-biopsy efficacy in predicting men who harbor aggressive prostate (8, 9). After biopsy, current National Comprehensive Cancer Network (NCCN) Guidelines recognize the potential value of the Oncotype Dx® Prostate and Promark® tissue-based tests for predicting the risk of Gleason grade 4 or non-organ confined disease at radical prostatectomy while the Prolaris® test improves the prediction of prostate-cancer specific mortality for men on active surveillance and the risk of biochemical recurrence after surgery or radiation (10). Thus, these biomarkers and genomic classifiers have an increasingly important role to select men for active surveillance versus definitive therapy. It is anticipated that novel and more informative biomarkers will be developed for clinical practice.

As the clinical integration of mpMRI and biomarkers continues to be refined and more widely used, there will be improved discrimination of patients who benefit from curative-intent surgery or radiation. In the future, it is likely that a smaller number of men will be treated for localized disease, but those receiving treatment will derive greater benefit from therapy.

## Non-traditional management strategies

A third major theme of the last decade that is likely to continue to evolve is the expansion of the role of both active surveillance and partial gland ablation strategies for management of localized prostate cancer. While we recognize that partial ablation is a form of treatment, we would argue that it avoids the main therapeutic extensions of whole gland therapy and should be considered independently from traditional surgery or radiation.

Level I evidence is now available supporting the use of active surveillance to monitor indolent disease. Hamdy et al. noted equivalent 10-year cancer specific mortality of less than 1.5% after active monitoring compared to surgery or radiotherapy in the randomized Prostate Testing for Cancer and Treatment (Protec T) trial (11). Similarly, promising outcomes have been noted in other prospective active surveillance registries (12). Furthermore, there is increasing interest in expanding active surveillance criteria in properly selected patients who will be monitored closely (13). Active surveillance is now recognized by the NCCN as a viable management strategy for very low risk, low risk and favorable intermediate-risk prostate cancer (10). In the coming years, it is likely that active surveillance protocols will become better standardized and further expanded outside of academic centers leading to an overall decline in patients requiring treatment.

Significant academic and clinical interest in partial gland ablation strategies have also had a meaningful impact on management of localized prostate cancer and we anticipate growth in the coming years. While there is a range of emerging ablative modalities, cryotherapy has the most robust focal ablative data. Analysis of the Cryo Online Data (COLD) registry demonstrated that a cohort of 317 men with low-risk prostate cancer undergoing focal therapy had comparable rates of 60-month biochemical recurrence but improved 24-month erectile function when compared to similar men who underwent whole gland ablation (14). We have also noted that focal ablation is particularly promising for solitary anterior lesions as there is less risk of collateral damage to the urinary sphincter and neurovascular bundles during treatment (15). More recently, there has been growing interest in the use of high intensity focused ultrasound (HIFU) as an ablative medium following United States Food and Drug Administration (FDA) approval as a class II device for ablation of prostate tissue (16). Emerging evidence on focal HIFU supports particularly high rates of continence and potency preservation (17).

In conclusion, we believe that there will be a meaningful decline in the treatment of localized prostate cancer, particularly by traditional surgery or whole gland radiation therapy. Negative sentiments surrounding screening are likely to decrease the number of men diagnosed with localized disease and early evidence suggests that there will be an upward stage migration to non-organ confined disease at the time of diagnosis. At the same time, we are optimistic about the integration of both mpMRI and markers to improve candidate selection for screening, biopsy and optimizing management strategies. Ultimately, these tools will improve detection of clinically significant disease and decrease detection and treatment of non-lethal disease. Finally, we believe the expansion of both active surveillance and partial ablative strategies have the potential to safely reduce the number of men requiring traditional treatment, particularly if they are applied in properly selected patients and performed by well-trained practitioners.
